# Regulatory emotional self-efficacy and anxiety in times of pandemic: a gender perspective

**DOI:** 10.1080/21642850.2022.2158831

**Published:** 2022-12-28

**Authors:** Esther Cuadrado, Manuel Rich-Ruiz, Tamara Gutiérrez-Domingo, Bárbara Luque, Rosario Castillo-Mayén, Joaquín Villaécija, Naima Z. Farhane-Medina

**Affiliations:** a Maimonides Biomedical Research Institute of Cordoba (IMIBIC), Cordoba, Spain; b Department of Psychology, University of Cordoba, Cordoba, Spain; c Department of Nursing, Pharmacology and Physiotherapy, University of Cordoba, Cordoba, Spain

**Keywords:** Regulatory emotional self-efficacy, anxiety, positivity, resilience, COVID-19 pandemic, gender

## Abstract

**Objectives:**

The COVID-19 pandemic and resultant lockdown and containment measures have instigated substantial changes in our daily lives and have affected many people’s mental health. This paper reports two studies exploring gender-based differences with regard to the impact of COVID-related confinement on individuals’ self-efficacy to regulate negative emotions (RESE-NE) and anxiety.

**Methods:**

Study 1 (cross-sectional; 269 participants; 52% women) explored the evolution of RESE-NE and anxiety. To this end, participants assessed their status at two time points: a retrospective assessment of the period before confinement in Spain, and a current assessment during confinement. Study 2 (longitudinal; 114 participants; 72.2% women) explored the evolution of the variables by adding a post-confinement time point and analyzed the mediating role of RESE-NE in the positivity–anxiety and resilience–anxiety relationships.

**Results:**

The results confirmed that: (a) RESE-NE decreased and anxiety increased more among women than among men during confinement (Study 1); (b) women recovered their pre-pandemic levels of mental health more slowly than did men following confinement; and (c) the mediating role of RESE-NE could be observed in the two relationships under analysis.

**Conclusion:**

In practical terms, the research highlights the need to pay special attention to women undergoing mental health interventions related to the COVID-19 pandemic, as well as to the differential burden that the pandemic may entail for men and women and to the contrasting social roles traditionally attributed to them. From the gender differences identified, it is possible to infer how stereotypes and social roles influence the behavior and mental health of men and women, leading them to cope differently with stressful situations such as confinement.

As the World Health Organization (WHO, 2020b) has highlighted, the coronavirus (COVID-19) pandemic has had an impact on individuals’ mental health. Given that the effects of pandemics and disease outbreaks can differ between men and women, the WHO (2020b) has called for analyses that utilize a gender perspective regarding the adverse health impacts, as well as the social and economic consequences of COVID-19, to examine men’s and women’s contrasting experiences. Unfortunately, at present, data disaggregated by sex are limited, rendering it difficult to analyze the gender-related impacts of COVID-19 as well as the development of appropriate responses. Medical research has also frequently looked at women ‘as if they were men,’ ignoring their problems, specific diseases, complaints, discomfort, and more (Valls-Llobet et al., 2007). However, it is important to analyze gender differences and attempt to understand their causes when carrying out psychosocial interventions adapted to both genders.

Along with a widespread fear of COVID-19, the changes produced in our daily lives due to a range of restrictions and confinement (e.g. working from home, homeschooling, social isolation) have affected mental health (WHO, 2020b). Confinement has necessitated homeschooling and the additional care of dependent people whose vulnerability may have been exacerbated by the pandemic, while additionally compelling people to adapt their working lives. Given that domestic tasks and the care of dependent people are roles traditionally assigned to women, confinement may have had a differential impact on men and women. It is therefore essential to conduct a gender-disaggregated study on the impact of the pandemic and confinement on mental health.

Previous research has highlighted how the COVID-19 pandemic has caused anxiety symptoms among the general population, whether directly or via additional psychosocial factors (Alzueta et al., 2021; Cuadrado et al., 2022; Kumar & Nayar, 2021). Beyond anxiety and depression, a mental health–related variable that could be particularly affected by COVID-related confinement is self-efficacy to manage negative emotions (RESE-NE; Caprara et al., 2008). RESE refers to the extent to which individuals perceive that they can manage negative emotions, so this variable may be particularly relevant during a sensitive period such as confinement that could enhance individuals’ negative emotions (Zhang et al., 2020), especially if we take into account that emotional regulation has been conceived as a protective factor when coping with traumatic and stressful events (Tyra et al., 2021) and in facing the mental health repercussions of COVID-19 (Panayiotou et al., 2021; Riaz et al., 2021; Tyra et al., 2021). Nevertheless, to date, the impact of confinement and the pandemic on RESE-NE has remained relatively underexplored. To counteract the mental health problems resulting from the pandemic, prevention and psychological interventions such as tools for emotional well-being are key (Boden et al., 2021). Moreover, prevention and psychological interventions must consider the potentially differential impact of the COVID-19 pandemic on men and women.

## Overview of the article

The main objective of this article is to analyze the impact of confinement on RESE-NE and anxiety by carrying out a comparative analysis of men and women. The paper reports two studies. Study 1 was cross-sectional: it explored the evolution of men’s and women’s reported levels of RESE and anxiety before and during confinement. Participants assessed their status at two time points: a retrospective assessment of the period before confinement in Spain and a current assessment during confinement. Study 2 was longitudinal: it monitored the evolution of the variables one month after the end of confinement and explored the mediating role of RESE in the link between specific personal variables and anxiety.

This paper’s relevance lies in its gender-based analyses of the evolution of two mental health variables: anxiety and RESE-NE. Given that men and women have been affected differently by COVID-19 and the associated confinement, disaggregated gender analyses such as those presented here are essential. Moreover, no previous studies have focused on the evolution of RESE-NE during confinement and the pandemic, although this variable is a relevant protective factor against mental health problems (Bandura et al., 2003; Caprara et al., 2008). In response, this research addresses the important question of how RESE-NE is affected by confinement. The second study is relevant due to its longitudinal character, as it replicates the earlier analysis of the evolution of anxiety and RESE-NE by including a second measurement time after confinement—that is, during the de-escalation period. Longitudinal data regarding the mental health impact of confinement and the COVID-19 pandemic disaggregated by sex are still quite scarce. Previous (generally longitudinal) studies regarding the evolution of mental health during the pandemic included diverse measurements during and after the period of confinement. By contrast, our research measured the health variables under analysis not only during the confinement time but also after confinement, during the de-escalation period. Nonetheless, a typical issue in longitudinal studies is the mortality of the sample. In this regard, the first study, which was cross-sectional, is relevant in that it provides a larger and more heterogeneous and balanced sample regarding gender, thereby enabling the results to be more widely generalized. All the specific hypotheses will be presented later in the individual studies.

## Study 1

Anxiety symptoms have been identified as one of the psychological consequences of the COVID-19 pandemic on the general population (Alzueta et al., 2021; Cuadrado et al., 2022; Kumar & Nayar, 2021), but they do not affect men and women equally. Although some studies have reported similar levels of anxiety between women and men (Ozamiz-Etxebarria et al., 2020), or greater vulnerability to anxiety among men during the quarantine necessitated by the pandemic (Chen et al., 2021), the literature has generally shown larger increases in the prevalence and severity of anxiety symptoms among women compared to men (Cárdaba-García et al., 2021; del Río-Casanova et al., 2021; Essangri et al., 2021; Hawes et al., 2022; Ripoll et al., 2021). In the Spanish context, for instance, Cárdaba-García et al. (2021) have found that anxiety and depression symptoms were especially common among women at the beginning of Phase 0 of the de-escalation period following confinement. Also in the Spanish context, del Río-Casanova et al. (2021) have provided evidence that the mental health impact of COVID-19 was greater among women in the early stages of the pandemic. Moreover, the longitudinal research of Ripoll et al. (2021), which explored the evolution of different mental health variables during the confinement period in Spain, indicated that women consistently exhibited higher anxiety and depression symptoms than men throughout the confinement period. Similarly, Hawes et al. (2022) showed that adolescent women have been particularly vulnerable to mental health problems during the COVID-19 pandemic, manifesting increases in depression and anxiety symptoms. Moreover, by using a more specific sample composed of medical students, Essangri et al. (2021) found that being female was a risk factor for severe symptoms of anxiety and other mental health outcomes during the early stage of the pandemic.

These discrepancies may be due to differences in gender socialization, specifically that women are traditionally more likely than men to care for dependent people and carry out household tasks, causing them to encounter an additional stressor. Consequently, some authors have focused on the cultural definition of gender roles to explain the contrasting levels of psychological distress experienced by men and women (Valls-Llobet et al., 2007; Zunzunegui et al., 1998). Although men are gradually becoming more involved in the private sphere, such as caring for dependent people and undertaking domestic tasks, the ‘double working day’ continues to be attributed to women in particular, inevitably affecting their psychological health as a result (Cannuscio et al., 2002; Griffin et al., 2002; Valls-Llobet et al., 2007).

RESE, which includes perceived self-efficacy to manage negative emotions (RESE-NE) and to express positive affect (Caprara et al., 2008), is particularly relevant in the COVID-19 context. This is because confinement has been accompanied by increases in negative emotions (Zhang et al., 2020), and effective emotional regulation is a protective factor in the face of traumatic events and stressful situations (Tyra et al., 2021), including against COVID-19’s repercussions for mental health (Panayiotou et al., 2021; Riaz et al., 2021; Tyra et al., 2021). The extent to which individuals perceive that they can manage negative emotions may thus be a particularly relevant variable during a sensitive period such as confinement.

Nevertheless, the evolution of RESE has yet to be explored in the pandemic crisis context. In this sense, one may hypothesize that particularly stressful and negative events like the pandemic and associated confinement, which plunge individuals into social isolation and compromise their well-being, affect not only their emotions, but also the efficacy with which they perceive they can manage the resulting negative emotions. Moreover, the potential decrease in RESE-NE caused by the negative impact of confinement on individual well-being may increase individuals’ stress and anxiety levels, as it has been observed that RESE is a protective factor against mental health problems (Bandura et al., 2003; Caprara et al., 2008). Given that no previous studies have explored the potential impact of confinement on RESE-NE, this research is thus highly relevant.

Long-established gender roles in Western societies also attribute the expression of internalizing negative emotions such as anxiety more to women, and the expression of externalizing negative emotion such as anger more to men (Eisenberg et al., 1996; Else-Quest et al., 2006). The roles that society expects women and men to assume inevitably influence both groups’ mental health and behavior, and therefore may explain the gender differences usually found in RESE-NE. As Bandura (1991) explains, one of the powerful antecedents of self-efficacy is the extent to which people whom we deem relevant express to us that we can perform a specified task. Thus, if society expects that women are unable to adequately regulate and manage their negative emotions, they will be affected by this social expectation and probably exhibit lower RESE-NE as a result.

Indeed, gender differences in RESE-NE have already been studied by Caprara et al. (2003), who found that men tend to enter adulthood with a stronger sense of regulatory emotional self-efficacy in managing negative affect than do women, although at older ages, they exhibit a weaker sense. Subsequently, several studies have corroborated the existence of gender differences in RESE (Alessandri et al., 2009; Caprara et al., 2008, 2013; Caprara & Steca, 2007), consistently showing lower levels of RESE-NE among women. Therefore, if there are gender differences in RESE-NE under typical circumstances, one can expect to find such differences within the confinement context as well.

**Hypothesis 1:** A greater increase in anxiety and a greater decrease in RESE-NE are expected during confinement among women than among men.

### Methods

#### Design and procedure

A retrospective cross-sectional and correlational design was applied by distributing anonymous online questionnaires. Due to the specific conditions at the time of the study, sampling was non-probabilistic, via snowball sampling (Goodman, 1961): A link to the questionnaire containing all the study variables was distributed by the research group and some students during lockdown via a range of social networks, although primarily through WhatsApp. Both a response to the questionnaire and further questionnaire dissemination were requested. Participants were asked to respond to the psychosocial variables by assessing their status at two time points: a retrospective assessment of the period before confinement, and a current assessment during confinement. The study, which was conducted in Spain, proceeded in accordance with the ethical principles for medical research involving human subjects of the World Medical Association of the Declaration of Helsinki (WHO, 2001) and received the approval of the human research ethics committee of the University of Córdoba through code CEIH-22-4. Informed consent was obtained before participants began to complete the survey. Prior to giving their consent, participants were informed of the objectives of the study and that their participation was voluntary, that their anonymity was guaranteed, and that they could withdraw from the study at any time. Nevertheless, at the end of the questionnaire, they were informed that, to be able to monitor the study variables and to evaluate their evolution over time, we needed them to answer a similar questionnaire a few months later. To do this, we asked the participants who wished to do so to provide us with their emails so that we could contact them in the future. They were informed that these emails would not serve to identify them, but simply to be able to link the data from the first survey with the second, without it being possible to use for other purposes, and being deleted from the database immediately after having linked their data from both surveys.

Before completing the questionnaire, participants were informed that the study was oriented to people residing in Spain. They were then asked to complete the questionnaire only if they reside habitually in Spain and to disseminate the questionnaire uniquely to their social networks who resided in Spain. Moreover, one of the demographic variables assessed their place of residence. No participants responded that they lived outside of Spain, so no participants were eliminated from the data due to their place of residence.

Almost all data were collected in late April 2020, between April 25 and April 30 (81.4%), and the rest of respondent completed the questionnaire between May 1 and May 21. All data collection was thus performed during confinement, which started on March 14 and did not end before June 21. Thus, the participants responded to the questionnaire about 4–8 weeks after the starting point of the confinement and in a period of decreased of the COVID-19 cases and deaths.

#### Measures

To fully describe the study sample, participants were asked if they were women or men, as well as other relevant sociodemographic data, such as age, place of residence, employment status, if they have partner, if they take care of children, if they take care of a dependent person at home, and the number of people sharing the residence during the lockdown. After responding to the sociodemographic questions, they responded to the psychosocial variables discussed below.

*Self-efficacy for the regulation of negative emotions.* To measure the extent to which individuals felt able to regulate their negative emotions, all eight items (e.g. ‘How well can you get over irritation quickly for wrongs you have experienced?’) referring to the negative emotion factor of the Regulatory Emotional Self-Efficacy Scale (Caprara et al., 2008) were completed twice by participants within the same questionnaire. This was presented as a 7-point Likert scale, where 1 corresponded to ‘not well at all’ and 7 to ‘very well.’ Participants responded by expressing the extent to which they felt able to regulate their negative emotions both before confinement (i.e. first) and, at present, during confinement (i.e. second). Reliability was high (α_before_ = .93; α_during_ = .96).

*Anxiety.* Anxiety was assessed using the anxiety factor of the validated Spanish version of the Hospital Anxiety and Depression Scale (HADS;, Herrero et al., 2003), which was completed twice by participants within the same questionnaire, using the same terms for RESE-NE. Participants responded to the seven items (e.g. ‘Do you feel tense and wound up?’) on a 7-point Likert scale, where 1 expressed ‘no anxiety’ and 7 ‘high anxiety.’ They responded by expressing the extent to which they felt anxious both before confinement (i.e. first) and in recent weeks during confinement (i.e. second). Reliability was adequate (α_before_ = .78; α_during_ = .82).

#### Statistical analyses

A repeated-measures analysis (RMA) was performed to assess the evolution of RESE-NE and anxiety, with time used as a within-subject factor (two levels were introduced for each variable: before and during confinement). Sex was analyzed as a between-subject factor to explore the possible interaction between changes over time and sex. Bonferroni correction was conducted for pairwise comparisons to test differences between (a) before confinement and during confinement and between (b) men and women.

By computing an a-priori power analysis for Study One with G*power software, using an F-test as the test family and analysis of variance (ANOVA; repeated measures, within-between interactions) as the statistical test, and by entering ‘2’ as both the number of groups and as the number of measurements and 0.47 as correlation among repeated measures (the more restrictive correlation among repeated measure of our data), while asking for a small to medium effect size (0.18), the results showed an optimal total sample size of 110 participants, with a power of 0.95. Therefore, our sample size (269 participants) was sufficient for the analyses performed.

### Results

#### Sample description

The sample comprised 269 people (52% of whom were women) from Spain (age range = 18–77, *M* = 41.29, *SD* = 12.92). Most participants were from Andalusia (69.1%), followed by Castilla y León (18.6%), the Community of Madrid (3%), and other communities (9.3%). The sociodemographic characteristics of the sample are shown in Supplementary Table 1.

#### Evolution of the variables from before to during confinement: repeated-measures analysis

**Figure 1.**
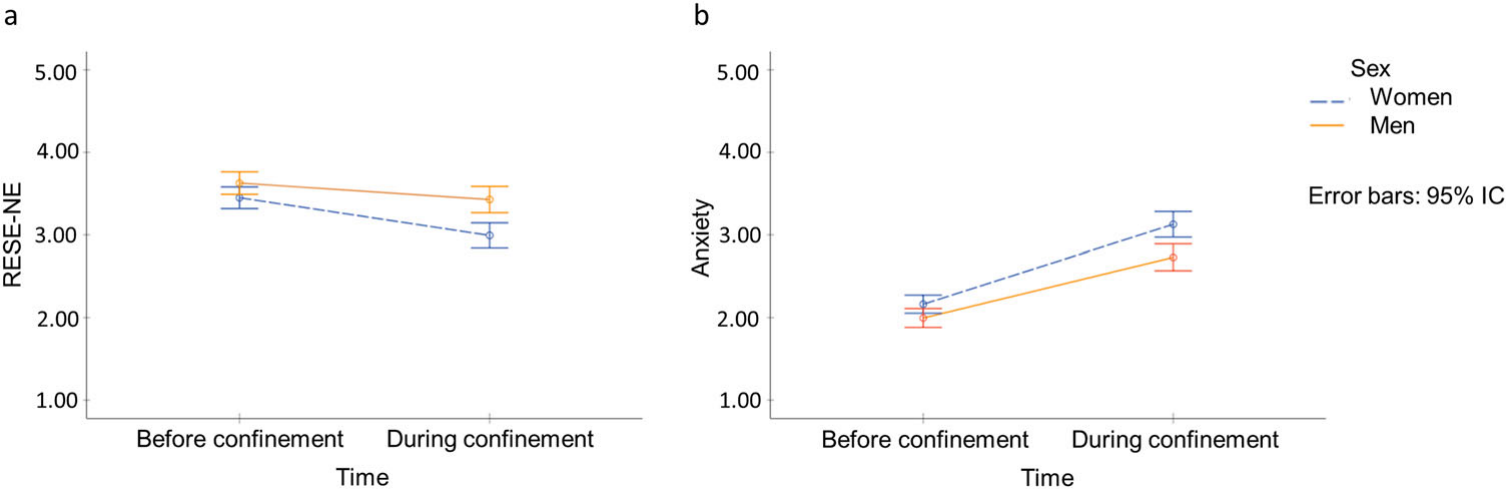
Changes in Regulatory Emotional Self-Efficacy of Negative Emotions (a) and Anxiety (b) Among Women and Men Over Time.

The results of the RMA (Figure 1) show the marginal estimated means for men and women regarding RESE-NE (Figure 1a) and anxiety (Figure 1b) at the two time points: before and during confinement.

For RESE-NE (Figure 1a), the RMA showed a main effect on time [*F*(1,267) = 54.56, *p *< .01, η^2^ = .17, observed power (OP) = 1.00], as well as an interaction effect on time by sex [*F*(1,267) = 8.35, *p *= .004, η^2^ = .030, O*P* = 0.82]. Pairwise comparisons showed: (a) significant differences between before and during confinement within the overall sample (Δ*M* = 0.328, *p* < .001; 95% IC = [0.241, 0.415]); (b) significant differences between before and during confinement among both women (Δ*M* = 0.456, *p* < .001; 95% IC = [0.335, 0.577]) and men (Δ*M* = 0.200, *p* = .002; 95% IC = [0.073, 0.326]); (c) marginal differences between women and men before confinement (Δ*M* = −0.178, *p* = .065; 95% IC = [−0.367, 0.011]); and (d) significant differences between women and men during confinement (Δ*M* = −0.434, *p* < .001; 95% IC = [−0.655, −0.214]). Our initial hypothesis for RESE-NE is thus supported: although it decreased significantly among both men and women, the drop was more pronounced among the latter.

For anxiety (Figure 1b), similar results were found, with a significant main effect on time [*F*(1,267) = 259.96, *p *< .001, η^2^ = .49, O*P* = 1.00], and an interaction effect on time by sex [*F*(1,267) = 4.77, *p *= .030, η^2 ^= .018, O*P* = 0.586]. The pairwise comparisons showed: (a) significant differences between before and during confinement within the overall sample (Δ*M* = −0.849, *p* < .001; 95% IC = [−0.953, −0.746]); (b) significant differences between before and during confinement among both women (Δ*M* = −0.964, *p* < .001; 95% IC = [−1.108, −0.821]) and men (Δ*M* = −0.734, *p* < .001; 95% IC = [−0.884, −0.585]); and (c) significant differences between women and men both before confinement (Δ*M* = 0.168, *p* = .038; 95% IC = [0.009, 0.326]) and during confinement (Δ*M* = 0.398, *p* = .001; 95% IC = [0.173, 0.622]). Therefore, with respect to anxiety, the hypothesis is supported: although anxiety increased for both men and women during confinement, anxiety increased more among women than among men.

### Discussion

The results of Study 1 showed how confinement during the COVID-19 pandemic was related to higher levels of anxiety among both men and women. These results are congruent with those of several other studies, which have estimated these rates to be in the range of 20%–30% (Chen et al., 2021; Hawes et al., 2022; Odriozola-González et al., 2022), albeit reaching as high as 63% when the risk of anxiety is considered (Cárdaba-García et al., 2021). However, although confinement was related to higher levels of anxiety among both men and women, in accordance with the previous literature (Cárdaba-García et al., 2021; del Río-Casanova et al., 2021; Hawes et al., 2022; Ripoll et al., 2021), the results of Study 1 also showed that these levels increased to a greater extent among women than among men. Such gender differences with regard to anxiety have frequently been explained by gender socialization and contrasting assumptions about caregiving tasks, which tend to be completed by women to a greater extent than by men, thereby providing an additional stressor that can increase women’s anxiety (Anyan & Hjemdal, 2018; Valls-Llobet et al., 2007; Zunzunegui et al., 1998). The impact of traditional gender roles may have been even more pronounced during confinement, and thus the well-known gender difference in the prevalence of anxiety remained at both time points of this evaluation.

Moreover, Study 1 showed how confinement owing to the pandemic reduced people’s levels of RESE-NE. These results are novel, because although several previous studies have explored the mental health impact of confinement on individuals (Alzueta et al., 2021; Cuadrado et al., 2022; Kumar & Nayar, 2021), as well as the protective role of functional and effective emotional regulation on the distress suffered due to the COVID-19 pandemic and associated lockdown measures (Panayiotou et al., 2021; Riaz et al., 2021; Tyra et al., 2021), the impact of confinement and the pandemic on RESE had yet to be explored. In this sense, our study highlights the lack of stability over time of RESE-NE in the particularly stressful and uncertain context of a health crisis, with emotional regulation proving to be affected by people’s negative experiences of confinement and the pandemic in general. This is an important result when we consider that, traditionally, RESE has been seen as a protective factor for mental distress (Bandura et al., 2003; Caprara et al., 2008). Moreover, congruently with previous research showing gender differences in RESE (Alessandri et al., 2009; Caprara et al., 2008, 2013; Caprara & Steca, 2007), the results of the present study indicate that although RESE-NE decreased significantly among both men and women, the drop was more pronounced among women. That is, during confinement, women perceived that they were less able than men to manage their negative emotions.

These differences between men and women must be considered according from a gender perspective. Regarding psychological health, female gender socialization is associated with a higher prevalence of distress (Valls-Llobet et al., 2007; Zunzunegui et al., 1998), as well as with lower validation and therefore externalization of negative emotions such as anger (Eisenberg et al., 1996; Else-Quest et al., 2006). The differential socialization that women tend to receive compared to men produces a kind of ‘emotional specialization,’ which reinforces feelings of sadness, fear, and guilt in learning what it means to be a girl (Jayme & Sau, 1996). In adulthood, the differential socialization of women is reflected in the assumption of gender roles that entail an overload of tasks and responsibilities—often focused on caring for other people—combined with the added burden of women’s presence in the labor market. This double presence/absence, which characterized much of the confinement period at home—as women were obligated to care for their dependents, while also teleworking—forces women to move from a culture of care to one of profit, internalizing the tensions that this entails (Comisión Interamericana de Mujeres & Organización de los Estados Americanos, 2020). All of these factors may explain the differences between men and women with regard to mental health, and must be considered both in psychological interventions and in government policies. Existing stereotypes and social roles influence the mental health and behavior of men and women, and could explain the different ways in which they face difficult and stressful situations, such as a pandemic and associated confinement. Consequently, they need to be addressed.

## Study 2

Previous longitudinal studies have shown that increases in anxiety are related to the duration of confinement due to COVID-19, and that, as the time of confinement increases, negative psychological symptoms increase significantly among the general population (Ozamiz-Etxebarria et al., 2020; Planchuelo-Gómez et al., 2020). Similarly, studies comparing countries where the pandemic has had different levels of impact and where contrasting confinement measures have been implemented have shown that people facing a more severe situation and stricter confinement policies have experienced relatively large increases in anxiety symptoms (Papandreou et al., 2020). However, some studies have indicated that following an initially negative effect on psychological well-being, several indicators (including anxiety) improved during the lockdown period (Ripoll et al., 2021). What, however, happens when the lockdown ends?

Given differences in mental health between women and men provoked by confinement, it is appropriate to analyze the long-term effects of confinement. This is because even after restrictive measures are removed, a situation of uncertainty can continue to exist, and with it, people’s emotional distress (Pieh et al., 2021; Pierce et al., 2021). Nevertheless, minimal research has explored the evolution of mental health during the pandemic, including examining the potential differences between before, during, and after confinement. Even less has done so from a gender perspective.

Admittedly, some studies have assessed the potentially differential evolution of mental health between men and women during confinement (Fenollar-Cortés et al., 2021; Salfi et al., 2020). Nevertheless, few studies have evaluated changes in mental health from the time of confinement to the subsequent period (although the present paper aims to do so). Research focusing on the evolution of mental health during confinement has shown that the mental health of women deteriorated to a greater extent than that of men (Ausín et al., 2021; Gamonal-Limcaoco et al., 2022), specifically in the form of higher levels of anxiety and other negative mental health symptoms. However, such gender differences diminished over time during the confinement period, with women manifesting a greater reduction in anxiety symptoms than men at follow-up (Fenollar-Cortés et al., 2021; Salfi et al., 2020). Nevertheless, what happens *after* confinement? Do differences remain between women and men? In their longitudinal study, Pierce et al. (2021) found different groups of trajectories for mental health evolution, showing that, in a period containing both confinement and post-confinement, the groups with the worst mental health evolution during the pandemic contain mainly women. Moreover, González-Sanguino et al. (2021) claim that it has not yet been possible to return fully to normality, and that attention should be paid to anxiety symptoms among women. Such a focus could include not only differences specific to women, but also (and especially) an analysis of what those differences mean for women’s lives and for the recovery of normality. Accordingly, we carried out a longitudinal study exploring the evolution of mental health before, during, and after confinement from a gender perspective, with the expectation that women would find it harder than men to recover their mental health.

To our knowledge, the impact of the pandemic on RESE has yet to be studied. Nonetheless, during the pandemic, negative emotions have increased (Zhang et al., 2020), and it has been found that functional emotional regulation predicts improved mental health outcomes (Riaz et al., 2021). Thus, one’s perception of being able to adequately manage negative emotions seems to be a relevant variable during the pandemic, especially when we consider that RESE has traditionally been seen as a protective factor against distress (Bandura et al., 2003; Caprara et al., 2008). Moreover, within the pandemic context, gender differences exist in RESE, with women generally feeling less able to cope with negative emotions than men (Alessandri et al., 2009; Caprara et al., 2008, 2013; Caprara & Steca, 2007). In this regard, Study 1 has already found that gender differences were maintained in the context of the COVID-19 pandemic: women claimed to be less able to manage negative emotions than men during confinement. Therefore, in light of the different social roles traditionally attributed to men and women, according to which women are expected to express their emotions in a specific way and to cope less well with negative emotions than men, we expect to find similar differences in the long term, with a slower recovery of confidence in managing negative emotions among women than among men.

**Hypothesis 1:** Once confinement is over, the return to normality in terms of RESE-NE and anxiety will be slower among women than among men.

On the other hand, certain psychosocial variables can sustain and influence emotional distress in a situation of confinement. Thus, an analysis of how some personal and regulatory variables may influence self-perceived anxiety after confinement seems relevant. Previous studies have suggested the decisive role of primary beliefs—both negative and positive—and dispositional states regarding the mental health consequences of the current pandemic. As noted by (Bozdağ, 2021), individuals who focused on the positive aspects of staying at home during confinement and regarded it as a responsible behavior, an opportunity, and a requirement to feel safe, experienced fewer psychological problems than people who did not have such a positive attitude. In this sense, both positivity and resilience seem to be particularly relevant personal variables that can play an important role in protecting one’s mental health in situations of uncertainty, such as confinement. A positive outlook about oneself and the future, and a positive adaptation to adversity (represented by positivity and resilience), should act as protective factors against anxiety in the face of the uncertain situation of confinement.

Positivity and RESE also appear to be associated, with a recent longitudinal study finding that the people who scored highest in positivity were the most confident in their abilities to regulate their emotions (Tabernero et al., 2021). Thus, it should be expected that individuals with high positivity feel better able to regulate their negative emotions during confinement than those with low positivity. In the same way, resilient individuals present positive outcomes despite facing negative events and challenges (Masten, 2011). Thus, it could be expected that individuals with a dispositional positive adaptation to situations of adversity feel particularly able to regulate their negative emotions during the uncertain situation of confinement.

Furthermore, all three attributes—positivity, emotional regulation, and resilience—have been related to mental health outcomes, such as anxiety and stress. Positivity has been related to distress in the context of COVID-19 (Yıldırım & Güler, 2021), and thus it should be expected that individuals with high dispositional positivity report lower anxiety levels after confinement than those with low dispositional positivity. Resilience has been conceived as a protective factor for mental health in negative and challenging situations, by predicting stress (Thomas & Zolkoski, 2020), distress and depression (Dowrick et al., 2008; Edward, 2005; Laird et al., 2019), and anxiety in the pandemic context (Anyan et al., 2020; Osimo et al., 2021). In this sense, resilience has been deemed a relevant target for psychological interventions to promote mental health during the pandemic (Ran et al., 2020). Thus, we expected that high levels of resilience (for both men and women) would predict low levels of anxiety after confinement. In addition, RESE has been shown to predict distress and depression (Bandura et al., 2003; Caprara et al., 2008); specifically, people who adequately self-regulate their negative emotions report low levels of anxiety and depression (Caprara et al., 2008). Therefore, we expected that individuals who feel able to adequately regulate their negative emotions during confinement would present relatively low levels of anxiety after confinement in comparison to individuals with lower levels of RESE-NE.

Consequently, according to the existing literature reviewed, it is possible to develop a hypothesis about the mediating role of emotional regulation in the relationship between positivity and resilience on the one hand and anxiety on the other. RESE has also been traditionally proposed as a mediating variable with effects on mental health (Dou et al., 2016; Yuan et al., 2018). Interestingly, this mediating role has recently been observed in the pandemic context in a cross-sectional study, confirming that RESE mediates the relationship between different personality factors (extroversion–introversion and neuroticism) and anxiety (Sui et al., 2021). In line with this, we propose through a longitudinal study that RESE-NE also acts as a mediator in the relationship between positivity and resilience on the one hand and anxiety on the other in the pandemic context. The additional personal variables explored (positivity and resilience) would influence RESE-NE during the confinement, which in turn would influence anxiety levels after confinement.

**Hypothesis 2:** RESE-NE mediates the relationship established between (a) positivity and anxiety and (b) resilience and anxiety.

### Method

#### Design and procedure

A retrospective longitudinal and correlational design was applied through a two-time distribution of a survey. The first measurement time was obtained using the data from Study 1. For the second measurement time, convenience sampling (Given, 2008) was used to recruit participants. All participants from Study 1 who agreed to participate in a follow-up study were contacted by email to request their participation in answering a second anonymous questionnaire, three months after the previous time-point evaluation, once confinement was over. Almost half of the sample of Study 2 (46.49%) completed the second questionnaire in late July 2020, between July 24 and July 31, and the rest of the respondents completed the questionnaire between August 1 and August 19. Thus, all the data collection was performed 5–8 months after the end of the confinement, in a period when the different restrictions remained, with some differences between the different Spanish areas that were generally related to the capacity in restaurants, bars, and other public places, and to the wearing of masks. Of the 269 participants in Study 1, 114 ultimately participated in Study 2. Thus, experimental mortality was 57.62%. As in Study 1, informed consent was given by participants prior to completing the online questionnaire.

#### Measures

*Positivity.* Positivity was measured with the Positivity Scale (Caprara et al., 2012), an eight-item (e.g. ‘I have great faith in the future') 7-point Likert-type scale ranging from 1 = ‘strongly disagree' to 7 = ‘strongly agree,' which evaluates the predisposition of people to estimate their life and their experiences from a positive perspective. Reliability for this scale was high (α = .83).

*Resilience.* Global resilience was assessed through the brief Spanish version (Serrano-Parra et al., 2013) of the CD-RISC-10 (Campbell-Sills & Stein, 2007). Participants responded to the 10 items (e.g. ‘I am able to adapt to change') on a 7-point Likert scale, where 1 was ‘not true at all' and 7 was ‘true nearly all the time.' The Cronbach’s alpha value for this scale was high (α = .90).

*Self-efficacy for the regulation of negative emotions.* As in Study 1, this was assessed by the eight items of the factor focused on negative emotions of the RESE scale (Caprara et al., 2008). The scale showed high reliability in the three time-point evaluations (before confinement α = .93, during α = .96, and after α = .88).

*Anxiety.* Participants’ anxiety was measured with the same instrument as in Study 1 (HADS, Herrero et al., 2003). Reliability was adequate (α = .78, .82, and .87 before, during, and after confinement, respectively).

#### Statistical analyses

As in Study 1, RMA was performed to assess the evolution of emotional regulation and anxiety, with time used as a within-subject factor (three levels were introduced for each variable: before, during, and after confinement). A between-subject factor was used for sex to explore the possible interaction between changes in both variables over time and participants’ sex. Bonferroni correction was conducted for pairwise comparisons to test differences between before, during, and after confinement, as well as between men and women.

A priori power analysis with G*power software was computed for Study 2, using an F-test as the test family and ANOVA (repeated measures, within-between interactions) as the statistical test and entering ‘2’ as the number of groups, ‘3’ as the number of measurements and ‘0.38’ as the correlation among repeated measures (the more restrictive correlation among repeated measure of our data) while asking for a small to medium effect size (0.18); this yielded an optimal total sample size of 102 participants, for a power of 0.95. Therefore, our sample size (114 participants) was sufficient for the analyses performed.

The Hayes SPSS Process Macro Test (Model 4) was conducted to test mediational analyses for Hypothesis 2. Resilience and positivity before confinement were introduced as independent variables, RESE during confinement was introduced as a mediating variable, and anxiety after confinement was used as the dependent variable.

A priori power analysis with G*power software was conducted, using an F-test as the test family and multiple linear regression as the statistical test, and entering ‘3’ as the number of predictors, while asking for a medium effect size (0.15); this yielded an optimal total sample size of 119 participants, for a power of 0.95. In the same analysis, while asking this time for a medium to large effect size (0.25), the optimal sample size was 73, with a power of 0.95. Therefore, our sample (114 participants) was quite small for the analyses performed for a medium effect size, but sufficient for analysis performed with a medium to large effect size.

### Results

#### Sample description

The sample comprised 114 people (72.2% of whom were women) residing in Spain (age range = 18–68, *M* = 39.87, *SD* = 12.06). The sociodemographic characteristics are shown in Supplementary Table 2.

#### Evolution of the variables from before to during confinement: repeated-measures analysis

**Figure 2.**
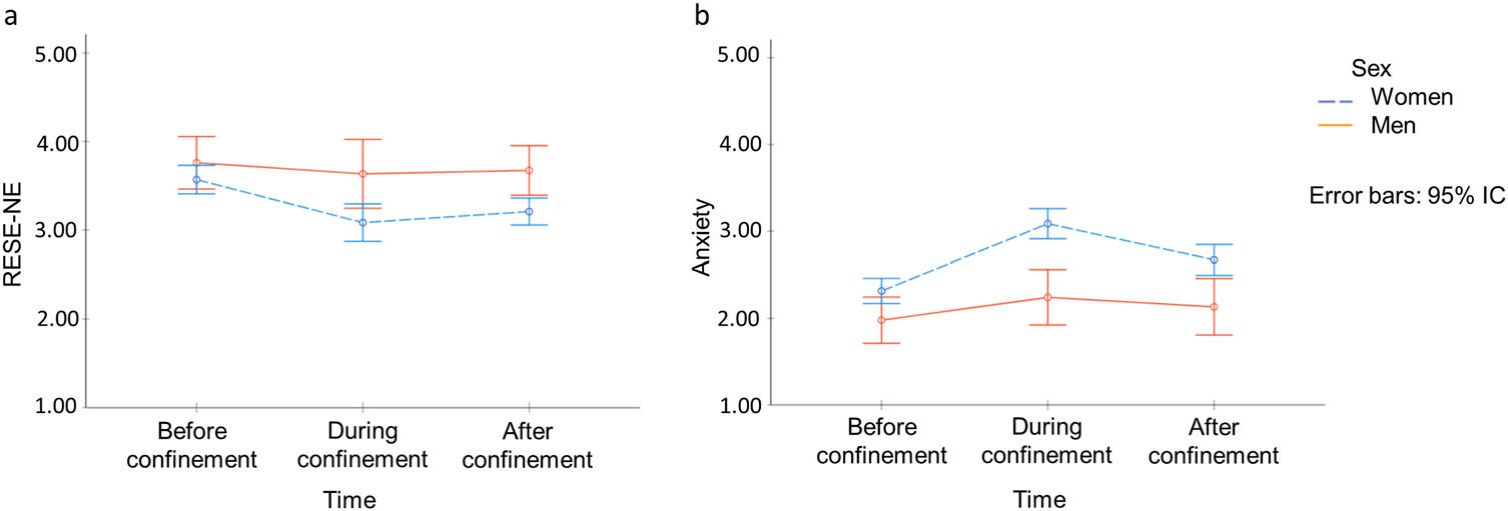
Changes in Regulatory Emotional Self-Efficacy of Negative Emotions (a) and Anxiety (b) Among Women and Men Over the Three Time Points.

Figure 2 shows the marginal estimated means for men and women regarding emotional regulation and anxiety at the three time-point evaluations (before, during, and after confinement).

For RESE-NE (Figure 2a), the RMA showed a main effect [*F*(2,224) = 7.43, *p *= .001, η^2 ^=  .62, O*P* = .93] and a marginal interaction effect on time by sex [*F*(2,224) = 2.64, *p *= .077, η^2 ^=  .023, O*P* = .51]. Furthermore, the pairwise comparisons showed: (a) significant differences between before and during confinement (Δ*M* = 0.305, *p* < .001; 95% IC = [0.114, 0.497]) and before and after confinement (Δ*M* = 0.224, *p* < .05; 95% IC = [0.040, 0.407]) in the overall sample; (b) significant differences between before and during confinement (Δ*M* = 0.486, *p* < .001; 95% IC = [0.303, 0.669]) and during and after confinement (Δ*M* = 0.361, *p* < .001; 95% IC = [0.186, 0.536]) for women; (c) no differences between time-point evaluations for men; and (d) significant differences between men and women during (Δ*M* = 0.551, *p* < .05; 95% IC = [0.108, 0.993]) and after (Δ*M* = 0.464, *p* < .05; 95% IC = [0.146, 0.783]) confinement, but not before.

For anxiety (Figure 2b), significant changes were found between before, during, and after confinement [*F*(2,224) = 14.03, *p* = .000, η^2 ^=  .11, O*P* = .99]. Additionally, an interaction effect on time by sex [*F*(2,224) = 3.42, *p *= .034, η^2 ^=  .030, O*P* = .63] was found. Moreover, pairwise comparisons showed: (a) significant differences between before and during confinement (Δ*M* = −0.517, *p* < .001; 95% IC = [−0.755, −0.280]), during and after confinement (Δ*M* = 0.263, *p* < .05; 95% IC = [0.28, 0.497]), and before and after confinement (Δ*M* = 0.255, *p* < .05; 95% IC = [0.015, 0.495]) in the overall sample; (b) significant differences among women between before and during confinement (Δ*M* = −0.771, *p* < .001; 95% IC = [−0.998, −0.544]), during and after confinement (Δ*M* = 0.416, *p* < .001; 95% IC = [0.191, 0.640]), and before and after confinement (Δ*M* = −0.356, *p* = .001; 95% IC = [−0.585, −0.126]); (3) no differences between time-point evaluations for men; and (4) significant differences between women and men before (Δ*M* = 0.337, *p* < .05; 95% IC = [0.034, 0.640]), during (Δ*M* = 0.844, *p* < .001; 95% IC = [0.481, 1.207]), and after confinement (Δ*M* = 0.539, *p* < .05; 95% IC = [0.168, 0.909]).

Hypothesis 1 is thus supported: RESE-NE and anxiety not only deteriorated to a greater extent among women throughout the phases of the study compared to men, but also apparently required more time to restore their baseline psychological state once confinement was over.

#### RESE-NE as mediator: mediational analyses

**Figure 3.**
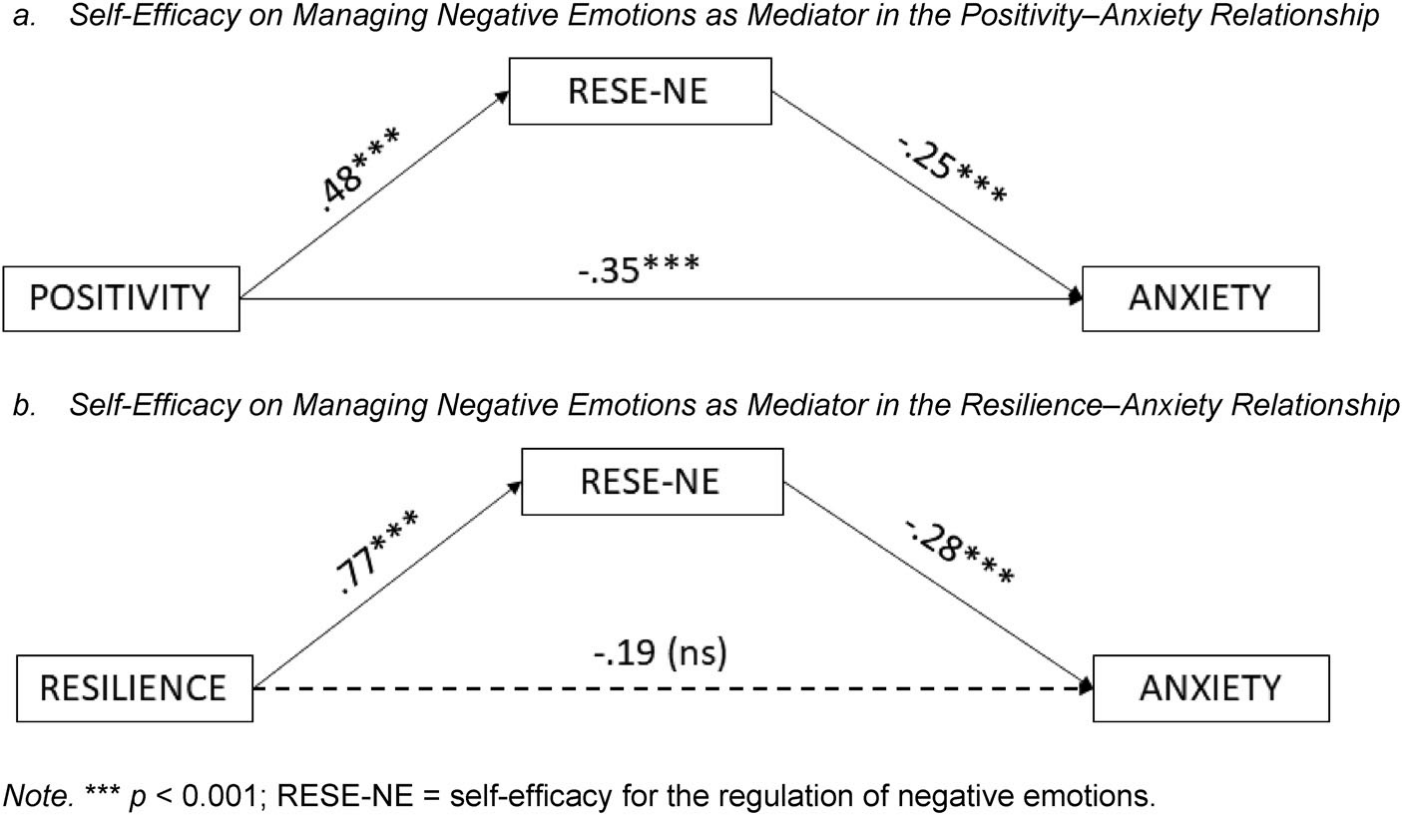
The Mediating Role of Self-Efficacy in Managing Negative Emotions.

Correlation analyses revealed that the variables used in the study were correlated in the expected direction (Supplementary Table 3). The mediating role of RESE-NE in the relationships established between (a) positivity and anxiety (*R*^2^ = 0.12; *F* (*df* = 1; 111) = 14.85, *p* < .001; completely standardized indirect effect (95% IC) = −.101 [−.201; −.025]) and (b) resilience and anxiety (*R*^2^ = 0.24; *F* (*df* = 1; 111) = 34.37, *p* < .001; completely standardized indirect effect (95% IC) = −.159 [−.288; −.043]) were confirmed, as can be observed in Figure 3. Therefore, Hypothesis 2 is supported by the results: negative emotional regulation mediates the relationship between (a) positivity and anxiety and (b) resilience and anxiety.

### Discussion

The results of Study 2 indicate that, during confinement, RESE-NE and anxiety not only deteriorated to a greater extent among women than among men, but also that once confinement was over, women required more time to return to normality. Women not only showed higher levels of anxiety and lower levels of RESE-NE than men during and after confinement, but also showed a worse evolution over time, requiring more time to restore their baseline level of self-efficacy than men, and not fully returning to their initial levels of anxiety after confinement.

This study’s findings are congruent with previous research showing that, among the general population, mental health did not return to normality following confinement (Pieh et al., 2021; Pierce et al., 2021), and more specifically with the study by Pierce et al. (2021) indicating that the groups with the worst mental health evolution mainly comprised women. Therefore, our data suggest a different time course of mental health between genders during the pandemic and highlight the importance of paying special attention to women, who seem to recover their previous condition more slowly than men. These results are particularly relevant considering that, as far as we know, few studies have evaluated the differential evolution of mental health among men and women between before, during, and after a period of confinement.

These gender differences may be explained by the influence of gender socialization. As explained earlier, the domestic sphere is traditionally attributed to women, including responsibilities like caring for dependent people and undertaking domestic tasks; furthermore, although men are now increasingly involved in the domestic sphere, differences remain between men and women. Alongside their typical responsibility for household tasks and caregiving, women today are also widely involved in the public sphere through their careers. This double role has inevitably added new stressors for women, which help explain the common differences that exist between men’s and women’s mental health (Anyan & Hjemdal, 2018; Valls-Llobet et al., 2007), as well as the greater anxiety levels experienced by women compared to men in particularly stressful situations such as confinement and other COVID-related restrictions (Comisión Interamericana de Mujeres & Organización de los Estados Americanos, 2020). Moreover, powerful stereotypes remain in society about how women should express their emotions: compared to men they are conventionally expected to manifest higher levels of emotional distress, to express negative emotions to a lesser extent, and to be less able to manage their emotions. These social expectations can compromise women’s self-efficacy regarding their ability to effectively manage their negative emotions (Luque et al., 2020) and explain why, in this study, women’s anxiety levels and RESE-NE recovered more slowly after confinement relative to men’s.

Regarding the role of RESE-NE as a mediator in the relationships between positivity and resilience on the one hand and anxiety on the other, the results of this study confirmed this mediating role. Thus, the results demonstrate that the more individuals have a positive outlook regarding themselves and the future, and the more resilient they are, the more they perceive themselves as being able to adequately regulate their negative emotions during confinement thus rendering them less affected by anxiety after confinement. These results are congruent with previous studies that have related positive dispositional beliefs (Bozdağ, 2021; Taroyan et al., 2020; Yıldırım & Güler, 2021) and resilience (Laird et al., 2019; Masten, 2011; Thomas & Zolkoski, 2020) to emotional regulation and mental health, as well as with research finding that RESE predicts distress both in normal contexts (Dou et al., 2016; Yuan et al., 2018) and in the more unusual pandemic context (Sui et al., 2021). Thus, these personal (positivity and resilience) and regulatory (RESE-NE) variables appear to play an important role as protective factors against anxiety in the face of the uncertain situation of a public health crisis such as COVID-19.

Positivity seems to play a particularly relevant role, because we found that it influences anxiety in two different ways: indirectly, through its effect on RESE-NE, and also directly. In this regard, and as (Bozdağ, 2021) has indicated, the relatively low levels of psychological problems among individuals who maintained a positive outlook despite staying at home due to the COVID-19 pandemic seem to recommend that mental health professionals focus on the development of positive feelings and thoughts in their interventions. In turn, this suggests that tools for emotional well-being may represent a useful intervention to improve people’s mental health in situations of confinement and pandemic.

## General discussion

The results of this research show how confinement during the COVID-19 pandemic was related to higher levels of anxiety and lower levels of self-efficacy in managing negative emotions within the overall population, and that such declines in mental health and self-efficacy were maintained even after the lockdown period ended. Moreover, these changes were particularly significant in the case of women, who not only showed greater anxiety and lower RESE-NE, but also seemed to need more time to return to normality. These results highlight the need to pay special attention to women in mental health interventions related to the pandemic, as well as to traditionally gendered social roles. Indeed, the ‘specialization’ of tasks with an overload for women may explain their greater difficulty in recovering their pre-pandemic levels of mental health. These results are particularly relevant considering that the evolution of mental health has tended to be studied during the confinement period, whereas few studies have paid attention to the subsequent period, and even fewer have done so from a gender perspective. Furthermore, to our knowledge, changes in the specific variable of RESE-NE have not previously been studied.

Additionally, the mediating role of self-efficacy in managing negative emotions in the relationship between certain personal variables (positivity and resilience) and anxiety has been confirmed, by highlighting the relevance of focusing on individuals’ perceived capacity to manage such emotions as a protective factor against anxiety in the face of the uncertainty of a public health crisis and associated confinement, which could be of value to prevention and psychological interventions. Once again, although the relationships between these individual predictors and mental health during the pandemic have already been studied, their relationships with mental health together with the specific mediation explored in this study are a novel contribution of this paper.

### Implications

The differences found between women and men in terms of RESE-NE and anxiety suggest that the negative psychological consequences of confinement during the COVID-19 pandemic were greater among women than among men. Both RESE-NE and anxiety increased to a greater extent among women than among men, and the former’s recovery was also slower. This different time course of mental health between genders during the pandemic highlights the relevance of paying special attention to women for COVID-related psychological interventions and prevention. Moreover, considering that high RESE-NE is a protective factor for mental health, it is necessary to focus on this variable in future interventions, particularly for women, as this research has shown that RESE-NE declined more significantly among this group during the pandemic.

Furthermore, the results of Study 2 regarding the mediating role of RESE-NE in the relationships between certain personal variables (positivity and resilience) and anxiety highlight the importance of promoting interventions oriented to helping individuals improve their self-efficacy. Doing so will enable people to better regulate negative emotions and thereby protect their mental health in the pandemic context. This is because it is easier to alter self-regulatory variables in comparison to positivity and resilience, which, despite being susceptible to change through interventions, tend to remain more stable over time.

Although the small sample size of Study 2 precluded us from exploring gender differences in the mediating model, the results are relevant from a gender perspective. Indeed, considering that they suggest RESE-NE is a relevant predictor of anxiety and a mediator of the relationships between positivity and resilience on the one hand and anxiety on the other, and given the gender differences in RESE, psychological interventions oriented to improving self-efficacy for emotional regulation could be particularly relevant for women to maintain or improve their psychological well-being during the COVID-19 pandemic. Additionally, it would be worth developing psychosocial interventions that focus on deconstructing traditional gender stereotypes about mental health, emotional management, and gender roles, as well as policies that favor the equal representation of men and women in the domestic and caregiving spheres. Finally, a ‘de-specialization’ of traditional roles is necessary, allowing both sexes to make compatible the tasks derived from the public and private spheres. After all, these two quite different spheres require individuals to invest considerable time and effort, potentially limiting their life and well-being if the responsibility of taking care of both spheres of life rests with only one person.

### Limitations and future research

One limitation of this research is related to the differential proportion of women in Study 2, who showed a significant drop from Study 1, although this did not prevent the results from supporting the initial hypotheses. Another limitation is the relatively small sample size of the longitudinal study, necessitating that the results of the moderation analyses be interpreted with caution. Another limitation related to the sample size in the longitudinal sample stems from the fact that the limited sample made it impossible to examine gender differences in the predictive model. It would be helpful for future studies to explore potential gender differences with regard to the moderating role of RESE, especially considering that gender differences were found in both the mediator and the dependent variable (anxiety) in the present research. Accordingly, future studies should aim to replicate the results of this research to test whether the gender differences, the relationships between variables, and the mediating role of RESE identified here are maintained in other countries, within other social contexts, and with larger samples.

Another limitation pertains to the use of online questionnaires for data collection, as individuals who do not have access to such technologies are easily excluded. Another limitation is also derived from the sampling method used. Although the questionnaires were disseminated via social networks, no methods were used to determine whether the same participants may have responded several times the questionnaires. Even if the possibility of one respondent responding several time is remote because no incentives where given for completing the questionnaires, which, moreover, took quite long to answer (with 30 items more all the demographic questions for Study one, and 30 items more all the demographic questions for Study two), this lack of control remains a limitation of the study. Nevertheless, we ensured that no duplicate data exist by eliminating all duplicate entries in the dataset. In the same way, we analyzed the sociodemographic data and no one of the responses offered by the participants in all the sociodemographic questions were the same as those of other participants; in each case some difference existed at least for one variable (sex, age, residence place, employment status, partner, children, dependent person at home, or number of people sharing the residence during the lockdown) between the different participants. This exploration of the data allowed us to deduce that no participants responded twice to the questionnaire.

Another limitation is the fact that measurement of the studied variables for the pre-confinement time was done afterward, by asking the participants, whilst in lockdown, to recall their anxiety and RESE levels before confinement. It must be acknowledged that social desirability effects could have influenced the results. In this sense, the generally flatter trend across time in the RESE variable found for men in comparison to women could be due to men being less willing to admit to being unable to manage their emotions, due to gender socialization, which usually orients men toward not admitting negative emotions that could be interpreted as a weakness (as sadness, for example) and toward being ‘strong,’ while in contrast it is generally more permissible for women to show their emotions than men. The literature thus confirmed that women usually express emotions like sadness and affection more frequently than men (but not pride, more expressed by men), which is coherent with the argument that the differences in gender expression are due to stereotyping and acquired through gender socialization rather than due to genetic processes (Brebner, 2003).

No data were collected about socioeconomic status and ethnicity, two relevant variables that merit consideration in studies analyzing COVID-19’s different impact by gender. Vulnerable communities have been disproportionately affected by COVID-19, including its adverse psychosocial and biological impacts (Bhaskar et al., 2020; World Health Organization WHO, 2020a). Certainly, belonging to a vulnerable group (e.g. having a low socioeconomic background or being part of an ethnic minority) and at the same time being a women may be a factor that exacerbates the negative impacts of COVID-19. Therefore, future research should analyze how gender interacts with other sociodemographic factors that shape vulnerability to COVID-19 (such as socioeconomic status and ethnicity) and thereby affects mental health.

Future research could also study the impact of current containment restrictions and the possible post-pandemic consequences for the anxiety and emotional regulation of women and men, including how they manage their lives. At the same time, there is a need to continue to explore gender differences to ascertain the potentially long-term impact of this public crisis on mental health, as women are expected to recover more slowly than men, a discrepancy that poses additional negative consequences. Finally, in addition to considering positivity and resilience, other relevant variables that may influence emotional regulation and anxiety should be studied, such as pandemic fatigue, perceptions of the effectiveness of protective measures, and risk perceptions of COVID-19 for health and economic status.

## Conclusions

This research confirms the adverse psychological effects of confinement and the COVID-19 pandemic in general, by adopting a gender perspective, as requested by the WHO (2020b). In particular, the results highlight the need to pay special attention to women, whose mental health seems to have been more significantly affected by the public health crisis and who recover their pre-pandemic mental health status more slowly than do men. (Nevertheless, it is necessary to recognize that this research has only considered anxiety and RESE-NE, and there may be other variables, not included here, which could indicate worse experiences among men than among women.) We argue that gender socialization is a possible factor behind these differences between men and women, and thus suggest that interventions oriented toward improving women’s mental health during the pandemic pay special attention to this aspect. Moreover, our results indicate that certain personal (positivity and resilience) and regulatory (RESE-NE) variables predict anxiety. Given that individuals’ perceived abilities to manage negative emotions play a mediating role and can be more easily changed by interventions than can personal attributes, interventions oriented toward improving people’s mental health status during the pandemic should therefore consider this variable.

## Supplementary Material

Supplemental Material

## Data Availability

The data are available at Cuadrado, Esther (2022), ‘Covid-Gender-Longitudinal’, Mendeley Data, V1, doi:10.17632/c4t5v3b9j4.1.

## Open Scholarship

This article has earned the Center for Open Science badges for Open Data and Open Materials. The data and materials are openly accessible at https://osf.io/epbm4/ and https://osf.io/epbm4/.
