# Supplementary material for: Regulatory emotional self-efficacy and anxiety in times of pandemic: a gender perspective
Source: Health Psychol Behav Med. 2022 Dec 28;11(1):2158831. doi: 10.1080/21642850.2022.2158831 (PMC9809367; doi:10.1080/21642850.2022.2158831)
Supplement: Supplemental Material [file RHPB_A_2158831_SM2555.zip › SupplTable1.docx]

| **Supplementary Table 1**  *Sociodemographic characteristics of the sample in women and men and statistical differences between both sexes* | | | | |
| --- | --- | --- | --- | --- |
|  | Total  (*N* = 269) | Women  (*n* = 140) | Men  (*n* = 129) | *Statistical significance* |
| Employment status, *n* (%) | | | | |
| Retired | 19 (7.1%) | 9 (6.4%) | 10 (7.8%) | *χ^2^* = 9.43, df 4, *p* = . 051 |
| Employed | 179 (66.5%) | 84 (60%) | 95 (73.6%) |  |
| Unemployed | 31 (11.5%) | 22 (15.7%) | 9 (7%) |  |
| Student | 38 (14.1) | 23 (16.4%) | 15 (11.6%) |  |
| Home care | 2 (0.7%) | 2 (1.4%) | 0 (0%) |  |
| Participants reporting having a partner, *n* (%) | | | | |
| Yes | 218 (81%) | 113 (80.7%) | 105 (81.4%) | *χ^2^* = 0.20, df 1, *p* = .887 |
| No | 51 (19%) | 27 (19.3%) | 24 (18.6%) |  |
| Participants reporting having to take care of children, *n* (%) | | | | |
| Yes | 146 (54.3%) | 76 (54.3%) | 70 (54.3%) | *χ^2^* = 0, df 4, *p* = .997 |
| No | 123 (45.7%) | 64 (45.7%) | 59 (45.7%) |  |
| Participants reporting having to take care of dependent person at home, *n* (%) | | | | |
| Yes | 17 (6.3%) | 9 (6.4%) | 8 (6.2%) | *χ^2^* = 0.06, df 1, *p* = .939 |
| No | 252 (93.7) | 131 (93.6%) | 121 (93.8%) |  |
| Number of people sharing the residence during the lockdown, *M* (SD) | 3.22 (1.13) | 3.29 (1.18) | 3.16 (1.07) | *t*(265) = .949, *p* = .344 |
