# Supplementary material for: Regulatory emotional self-efficacy and anxiety in times of pandemic: a gender perspective
Source: Health Psychol Behav Med. 2022 Dec 28;11(1):2158831. doi: 10.1080/21642850.2022.2158831 (PMC9809367; doi:10.1080/21642850.2022.2158831)
Supplement: Supplemental Material [file RHPB_A_2158831_SM2555.zip › SupplTable2.docx]

| **Supplementary Table 2**  *Sociodemographic characteristics of the sample in women and men and statistical differences between both sexes* | | | | |
| --- | --- | --- | --- | --- |
|  | Total  (*N* = 114) | Women  (*n* = 88) | Men  (*n* = 26) | *Statistical significance* |
| Employment status, *n* (%) | | | | |
| Retired | 6 (5.3%) | 4 (4.5%) | 2 (7.7%) | *χ^2^* = 5.352, df 4, *p* = .253. |
| Employed | 70 (61.4%) | 50 (56.8%) | 20 (76.9%) |  |
| Unemployed | 21 (18.4%) | 18 (20.5%) | 3 (11.5%) |  |
| Student | 16 (14%) | 15 (17.1%) | 1 (3.9%) |  |
| Home care | 1 (0.9%) | 1 (1.1%) | 0 (0%) |  |
| Change of employment status after confinement *n* (%) | | | | |
| Same employment | 48 (42.1%) | 36 (40.9%) | 12 (46.1%) | *χ^2^* = 1.556, df 5, *p* = .907 |
| Adaptation to teleworking | 39 (34.2%) | 31 (35.2%) | 8 (30.7%) |  |
| Reduction of working hours or retribution | 5 (4.4%) | 4 (4.6%) | 1 (3.9%) |  |
| Job loss | 17 (14.9%) | 14 (15.9%) | 3 (11.5%) |  |
| Increase in working hours | 2 (1.8%) | 1 (1.1%) | 1 (3.9%) |  |
| Leave of absence | 3 (2.6%) | 2 (2.3%) | 1 (3.9%) |  |
| Participants reporting having a partner, *n* (%) | | | | |
| Yes | 82 (71.9%) | 61 (69.3%) | 21 (80.8%) | *χ^2^* = 1.303, df 1,  *p* = .254 |
| No | 32 (28.1%) | 27 (30.7%) | 5 (19.2%) |  |
| Participants reporting having to take care of children, *n* (%) | | | | |
| Yes | 60 (52.6%) | 42 (47.7%) | 18 (69.2%) | *χ^2^* = 3.722, df 1, *p* = .054 |
| No | 54 (47.4%) | 46 (52.3%) | 8 (30.8%) |  |
| Participants reporting having to take care of dependent person at home, *n* (%) | | | | |
| Yes | 4 (3.5%) | 2 (2.3%) | 2 (7.7%) | *χ^2^* = 1.741, df 1, *p* = .187 |
| No | 110 (96.5) | 86 (97.7%) | 24 (92.3%) |  |
| Number of people sharing the residence during the lockdown, *M* (SD) | 3.04 (1.12) | 2.99 (1.18) | 3.24 (1.07) | *t*(111) = -.989, *p* = .325 |
