# Supplementary material for: Regulatory emotional self-efficacy and anxiety in times of pandemic: a gender perspective
Source: Health Psychol Behav Med. 2022 Dec 28;11(1):2158831. doi: 10.1080/21642850.2022.2158831 (PMC9809367; doi:10.1080/21642850.2022.2158831)
Supplement: Supplemental Material [file RHPB_A_2158831_SM2555.zip › SupplTable3.docx]

| **Supplementary Table 3.**  *Pearson Correlation Matrix for the Studied Variables* | | | | | | | | |
| --- | --- | --- | --- | --- | --- | --- | --- | --- |
|  | 1 | 2 | 3 | 4 | 5 | 6 | 7 | 8 |
| 1. Positivity | - |  |  |  |  |  |  |  |
| 2. Resilience | .629^**^ | - |  |  |  |  |  |  |
| 3. Emotional regulation BC | .354^**^ | .418^**^ | - |  |  |  |  |  |
| 4. Emotional regulation DC | .343^**^ | .486^**^ | .711^**^ | - |  |  |  |  |
| 5. Emotional regulation AC | .568^**^ | .533^**^ | .589^**^ | .618^**^ | - |  |  |  |
| 6. Anxiety BC | -.261^**^ | -.158 | -.216^*^ | -.160 | -.262^**^ | - |  |  |
| 7. Anxiety DC | -.379^**^ | -.296^**^ | -.151 | -.508^**^ | -.327^**^ | .383^**^ | - |  |
| 8. Anxiety AC | -.400^**^ | -.300^**^ | -.196^*^ | -.394^**^ | -.446^**^ | .372^**^ | .508^**^ | - |
| Mean | 3.64 | 3.74 | 3.61 | 3.21 | 3.32 | 2.24 | 2.89 | 2.55 |
| Sd | 0.73 | 0.64 | 0.76 | 1.02 | 0.74 | 0.69 | 0.89 | 0.86 |
| *Notes.* * *p* < 0.05; ** *p* < 0.01. BC = before confinement; DC = during confinement; AC = after confinement. | | | | | | | | |
